# Novel Anticoagulants and Hip Fractures in the Elderly

**DOI:** 10.7759/cureus.23020

**Published:** 2022-03-10

**Authors:** George Matheron, Imani Looby, Mehdi Khan, Muhammad A Fazal

**Affiliations:** 1 Trauma and Orthopaedics, Royal National Orthopaedic Hospital, London, GBR; 2 Trauma and Orthopaedics, Barnet Hospital, London, GBR

**Keywords:** neck of femur fractures, 30-day mortality, geriatric hip fracture, elderly trauma, direct oral anticoagulant therapy

## Abstract

Introduction

Early surgery is recommended in hip fractures to reduce morbidity and mortality. Surgery is often delayed in patients on novel direct oral anticoagulants (DOACs). The purpose of our study was to investigate the impact of DOACs on patients with hip fractures.

Methods

A retrospective comparative analysis was performed. A total of 766 patients presented with neck of femur fractures in the study period. Patients under the age of 60, those managed conservatively and those on alternative anticoagulation (including warfarin, clopidogrel and aspirin) were excluded. Forty-seven (6.1%) patients were on DOACs, to which a group of 47 patients was matched for age, gender, fracture type and intervention to minimise confounding. Primary outcome data on time to surgery (TTS), pre-/postoperative haemoglobin, haemoglobin drop, length of stay (LOS) and 30-day mortality were collected, as well as secondary outcome data on blood transfusion and wound complications. The Charlson Comorbidity Index (CCI) was calculated for all patients.

Results

The mean Charlson Comorbidity Index was significantly increased in the DOAC group (p<0.0001). The mean time to surgery was 49.5 hours in the DOAC group versus 31.3 hours in the control group (p=0.0002). Haemoglobin drop for DOAC patients was 16.9 g/L and 15.9 g/L for control patients (p=0.6). Similarly, no significant increase in transfusion was required (p=0.74). Six DOAC patients and two control group patients died within 30 days of surgery (p=0.13). Wound complications were seen in five (10.6%) patients on DOAC and two (4.2%) patients in the control group (p=0.02).

Conclusion

The results demonstrate statistically significantly higher comorbidities, delay in surgery and higher wound complications in patients on DOAC but no significant difference in haemoglobin drop, blood transfusion and mortality.

## Introduction

Hip fractures are the commonest reason for acute orthopaedic hospital admission, with 67,302 cases in the UK as of 2019. Numbers are predicted to rise with the increasingly elderly population [1].

Timely surgery has been advocated with the aim of improving outcomes, with worsening morbidity and mortality, particularly beyond 48 hours [2]. Some debate remains around the effect of earlier surgery on outcomes such as 30-day mortality. However, time to theatre remains an important metric, being seen as an independent risk factor. Additionally, earlier surgery may help avoid unnecessary pain for the patient and lead to earlier rehabilitation and mobilization [3].

Many elderly hip fracture patients have multiple comorbidities, including a higher prevalence of cardiovascular disease, often requiring treatment with anticoagulant medications [4]. Novel or now more commonly called direct oral anticoagulants (DOACs) have seen ever-increasing use in this cohort, as they have been shown to have significantly lower major bleeding events than in warfarin users and require less frequent monitoring due to fewer interactions and larger therapeutic ranges [5].

However, compared to vitamin K antagonists, DOACs have less widely available reversal agents [6]. Perioperative management guidelines are less well established and therefore often vary between hospitals. However, in the absence of well-evidenced reversal protocols, it is commonplace to omit DOACs for 24-48 hours prior to surgery [7]. This poses ongoing challenges for trauma and orthopaedics departments worldwide, as they face increases in the elderly comorbid hip fracture admissions on DOACs.

Anticoagulants have been associated with delays in time to surgery (TTS), primarily due to the need for reversal and concern over bleeding complications [8,9]. Hence, it is important for surgeons to be aware of the effect of anticoagulation on management and outcomes. Here, our aim was to compare the effect of DOAC usage, versus not, on patient outcomes in elderly patients with hip fractures.

## Materials and methods

Study design

This is a retrospective comparative observational study of patients with local hip fractures presenting to a busy London general hospital between January 2018 and December 2019. The study aims to compare characteristics and outcomes between patients presenting with hip fractures on direct oral anticoagulants (DOACs) and those not anticoagulated.

Inclusion and exclusion criteria

Patients presenting with intracapsular or extracapsular hip fractures undergoing any type of surgery were included in the study. Exclusion criteria included patients under the age of 60; those on alternative anticoagulation including warfarin, aspirin and clopidogrel; and patients managed conservatively. Patients admitted on DOAC therapy were our main study group, and to reduce the effect of confounding, a matched patient of similar age, gender, fracture and type of surgery received were included in the control group. Following the identification of DOAC patients, the remaining operatively managed patients were individually matched according to the aforementioned criteria by the lead and senior author.

Data collection

Patient data were collected from the local hip fracture database, in which information on all patients admitted to the hospital with hip fractures are recorded. This database revealed 766 hip fractures across the study period. Electronic records were reviewed, including laboratory results and patient charts. The data extracted included standard demographic data for age and gender, as well as surgical management. Primary outcome data included information on time to surgery (TTS), preoperative and postoperative haemoglobin, haemoglobin drop, length of stay (LOS) and 30-day mortality. For the secondary outcomes, we included the need for blood transfusion and wound complications. The Charlson Comorbidity Index (CCI) was calculated for all patients.

Statistical analysis

Statistical analysis was performed using the GraphPad software. An unpaired t-test was used for any appropriate continuous data, including the Charlson Comorbidity Index, time to surgery, length of stay and change in haemoglobin. A chi-square test was used to calculate p-values for 30-day mortality and transfusion count.

## Results

Across the study period, 766 neck of femur fractures presented to our service, with 47 (6.1%) of patients on DOACs, to which 47 control-matched patients were included. Anticoagulation was spread across four main DOAC medications, with the majority of patients taking apixaban (n=39) and the remainder taking rivaroxaban (n=4), edoxaban (n=2) and dabigatran (n=2). The mean age was 84.9 years in the DOAC group and 84.2 years in the control group, representing no significant difference (p>0.5). Fracture type and surgery performed were similar for each group (Table 1). However, the DOAC group showed a significantly higher comorbidity burden as compared with the non-DOAC group. The mean Charlson Comorbidity Index was 7.87 for the DOAC group and 5.64 for the control group (p<0.05).

**Table 1:** Type of surgery in both direct oral anticoagulant and control group

	DOAC patients (n=47)	Control patients (n=47)
Cemented hemiarthroplasty	21	22
Dynamic hip screw	13	13
Total hip replacement	2	2
Long cephalomedullary nail	6	6
Short cephalomedullary nail	1	1
Uncemented hemiarthroplasty	4	3

The mean time to surgery was 49.5 hours in the DOAC group and 31.3 hours in the control group, following a normal distribution (p<0.05), whilst the 30-day mortality in patients on DOACs was 12.7% and in patients who were not on DOAC was 4.2% (p>0.05).

The mean haemoglobin drop for DOAC patients was 16.9 g/L and for the control group was 15.9 g/L (p>0.05). Seven (14.6%) patients required blood transfusion in the DOAC group, whilst six (12.5%) needed blood transfusion in the control group (p>0.5).

The mean length of stay in patients on DOACs was 14.6 days and in the control group was 18.1 days (p>0.5).

Wound complications, such as postoperative wound ooze, were seen in five (10.6%) patients on DOAC and no patients in the control group (p=0.02). However, no patients required reoperation for wound management (Table 2).

**Table 2:** Age and outcomes of patients on direct oral anticoagulants (DOACs) and control patients

		DOAC patients (n=47)	Control patients (n=47)	P-value (1s.f.)
Age	Mean (SD)	84.98 (7.04)	84.2 (5.84)	0.6
Charlson Comorbidity Index (CCI)	Mean (SD)	7.87 (1.79)	5.64 (1.66)	<0.0001
Time to surgery (TTS) (hours)	Mean (SD)	49.33 (13.35)	31.36 (27.77)	0.0002
Oozy wounds	Count (%)	5 (10.6)	0 (0)	<0.5
Haemoglobin change (g/L)	Mean (SD)	-16.98 (11.92)	-15.91 (9.05)	0.6
Blood transfusion	Count (%)	7 (14.6)	6 (12.5)	0.7
Length of stay (LOS) (days)	Mean	14.6	18.1	>0.5
30-day mortality	Count (%)	6 (12.7)	2 (4.2)	>0.5

## Discussion

Mortality from hip fractures is high, reaching 6.5% at 30 days and 30% at one year [1]. This high morbidity and mortality burden is often not attributable to the fracture alone and instead represents the complex medical, surgical, and rehabilitation needs of these patients [10].

In an ageing and increasingly comorbid population, we are likely to see increasing numbers of patients with hip fractures on DOACs. These patients, as in our study, have a greater comorbid burden, which may have impacts on management and recovery. In this context, it is important to explore any variables that can affect timely management and consequent outcomes.

We compared time to surgery, haemoglobin drop, the number of blood transfusions, length of stay, wound complications and 30-day mortality between a control group and a test group of patients on DOACs. There was no significant difference in haemoglobin drop, transfusion rate, length of stay and 30-day mortality, but there was a statistical significance in delay in surgery and a higher rate of wound discharge.

Time to surgery (TTS)

Within our centre, patients on DOACs are operated on >24 hours following the administration of the last dose, depending on renal impairment (assessed via creatinine clearance calculation). Recommencement occurs 24 hours postoperatively where deemed safe by the attending clinician. Findings regarding delay to theatre in DOAC patients have been variable, with some studies reporting increased risk of operations occurring beyond 48 hours from admission [11]. This is consistent with our findings that patients on DOACs had significant TTS delays compared to our control patients (49.3 hours and 31.4 hours, respectively). As a consequence, DOAC patients are less likely to meet the best practice tariff compared with those not on DOACs. However, other studies have found that time to surgery lies within the 36-hour target [12,13].

The challenge of patients presenting on new-style DOAC medications typically relates to the reversal of anticoagulant effects to allow for safe surgery, minimising bleeding risk. More traditional vitamin K antagonists such as warfarin have well-established reversal protocols with widespread experience of treating teams in the perioperative phase. In contrast, newer DOAC medications have less reliable anticoagulation monitoring, associated with widespread use being uncommon. Dabigatran, which remains the only anticoagulant with a specific reversal agent (idarucizumab) and high associated cost, sees limited use. Although perioperative management guidelines have been suggested, internationally accepted and utilized guidelines remain elusive [14]. It has therefore been suggested that DOAC patients may experience greater delays to surgery than those on more traditional vitamin K antagonists such as warfarin [11]. This may represent variability in the perioperative management of DOACs, as well as the need for individualised management of multiple comorbid patients. Therefore, it is important to ask what impact does theatre delay has on these patients.

Mortality

Delay in the surgical management of hip fractures has long been suggested as a cause for increased morbidity and mortality [15,16]. Early surgery is often advocated; however, there remains some debate on the definition of what constitutes ‘early’ hip fracture surgery [15,17,18]. Some authors advocate for surgery before 24 hours, although others suggest no effect of delay to surgery beyond 24 hours [19-21]. Meta-analysis has highlighted a significant increase in mortality and morbidity, such as pressure sores and pneumonia, in delays longer than 48 hours [22].

Our findings report no associations with longer time to surgery and 30-day mortality, suggesting that the current local regimen is safe and not associated with any significant effects on patient care and outcome. As there is no consensus on the minimum significant difference in surgical timing for patients on DOACs, surgery as early as is practically possible allowing for patient optimisation should be considered.

Haemoglobin drop and blood transfusion

Within our centre, red blood cell transfusion is commonly considered with haemoglobin levels of either <8 g/dL or <10 g/dL in symptomatic patients. We found no significant difference between patients who were anticoagulated against those who were not. This result is consistent with other authors, suggesting that there is no difference in bleeding complications between these cohorts. Therefore, it may be safe to minimise surgical delay in anticoagulated patients [13,23-25]. Instead, other factors such as fracture type and surgical type or technique may have effects on haemoglobin drop rather than simply anticoagulation management.

Length of stay

Length of stay is a commonly used metric for hospital management, helping guide the resources required to care for postoperative patients, as well as the consequent financial implications. Several studies have shown that time to surgery leads to longer postoperative stays [20-26]. However, Ho et al. found no relationship when controlling for confounders [27].

When looking more specifically at patients on DOACs, our study saw delays to theatre and increased rates of wound ooze; however, this did not translate to a statistically significant difference in length of stay. This is mirrored in a study by Leer-Salvesen et al. [24].

Wound complications

Across our cohorts, a significantly higher incidence of wound ooze was found in patients on DOACs; however, this was not associated with any need for surgical intervention. Other authors have found no significant difference and no increase in reoperation rates for patients on DOACs [13,28]. Alongside evidence that delay to surgery may increase the likelihood of surgical wound infection [29], it can be suggested that expediting surgery is prudent to minimise wound complications and reoperation rates.

Limitations

There are several limitations to our study. Its retrospective nature relies on the accurate recording of data by treating teams at the time of presentation. Furthermore, with a small sample size, small effects may not be notable that impact on the increasingly large hip fracture cohort on DOACs. Patients in both groups were also selected based on a few variables, mainly age and type of surgery. The management of multiple comorbid and often frail patients is complex, and therefore, our study may not highlight confounding factors affecting the outcomes of these patients in our cohort. Although pre- and postoperative haemoglobin tests are performed at standardised times (on admission and day 1 postoperatively), transfusion protocols may vary between clinicians and therefore may be difficult to compare across studies.

## Conclusions

In conclusion, it is clear that a significant anticoagulant burden remains for patients presenting with hip fractures on DOACs, with studies reporting conflicting results. The results in our cohort demonstrate that patients on DOACs were significantly more comorbid, with a longer time to surgery and more wound complications than non-DOAC patients. However, this does not translate to a significant difference in haemoglobin drop, transfusion rates, mortality or length of stay. As few studies focus on the effect of newer direct oral anticoagulants in this cohort, our data remains relevant in the understanding of this problem. Further studies of larger cohorts are still required to better understand the effects of specific DOACs over longer follow-up periods and assess the use of clear guidelines that can be used more ubiquitously to standardise management and allow for the prompt delivery of surgery.
